# Efficacy of Intraoperative Recurrent Laryngeal Nerve Monitoring During Thoracoscopic Esophagectomy for Esophageal Cancer: A Systematic Review and Meta-Analysis

**DOI:** 10.3389/fsurg.2021.773579

**Published:** 2021-11-03

**Authors:** Xinxin Wang, Haixie Guo, Quanteng Hu, Yongquan Ying, Baofu Chen

**Affiliations:** Department of Thoracic and Cardiovascular Surgery, Affiliated Taizhou Hospital of Wenzhou Medical University, Taizhou, China

**Keywords:** esophagectomy, esophageal cancer, recurrent laryngeal nerve monitoring, lymphadenectomy (LA), mini-invasive

## Abstract

**Background:** Recurrent laryngeal nerve paralysis (RLNP), a severe complication of mini-invasive esophagectomy, usually occurs during lymphadenectomy adjacent to recurrent laryngeal nerve. This systematic review and meta-analysis aimed to evaluate the efficacy of intraoperative nerve monitoring (IONM) in reducing RLNP incidence during mini-invasive esophagectomy.

**Methods:** Systematic literature search of PubMed, EMBASE, EBSCO, Web of Knowledge, and Cochrane Library until June 4, 2021 was performed using the terms “(nerve monitoring) OR neuromonitoring OR neural monitoring OR recurrent laryngeal nerve AND (esophagectomy OR esophageal).” Primary outcome was postoperative RLNP incidence. Secondary outcomes were sensitivity, specificity, and positive and negative predictive values for IONM; complications after esophagectomy; number of dissected lymph nodes; operation time; and length of hospital stay.

**Results:** Among 2,330 studies, five studies comprising 509 patients were eligible for final analysis. The RLNP incidence was significantly lower (odds ratio [OR] 0.33, 95% confidence interval [CI] 0.12–0.88, *p* < 0.05), the number of dissected mediastinal lymph nodes was significantly higher (mean difference 4.30, 95%CI 2.75–5.85, *p* < 0.001), and the rate of hoarseness was significantly lower (OR 0.14, 95%CI 0.03–0.63, *p* = 0.01) in the IONM group than in the non-IONM group. The rates of aspiration (OR 0.31, 95%CI 0.06–1.64, *p* = 0.17), pneumonia (OR 1.08, 95%CI 0.70–1.67, *p* = 0.71), and operation time (mean difference 7.68, 95%CI −23.60–38.95, *p* = 0.63) were not significantly different between the two groups. The mean sensitivity, specificity, and positive and negative predictive values for IONM were 53.2% (0–66.7%), 93.7% (54.8–100%), 71.4% (0–100%), and 87.1% (68.0–96.6%), respectively.

**Conclusion:** IONM was a feasible and effective approach to minimize RLNP, improve lymphadenectomy, and reduce hoarseness after thoracoscopic esophagectomy for esophageal cancer, although IONM did not provide significant benefit in reducing aspiration, pneumonia, operation time, and length of hospital stay.

## Introduction

Thoracoscopic radical esophagectomy is an important surgical approach in esophageal cancer, and lymphadenectomy during esophagectomy is necessary because lymph node metastasis is common in patients with esophageal cancer. Lymph nodes along the recurrent laryngeal nerve (RLN) is a common site of metastasis, and radical dissection of lymph nodes along bilateral RLNs is important for improving patient prognosis (1–6). However, injury to RLN, which usually occurs during lymphadenectomy, can lead to RLN paralysis (RLNP), a cause of hoarseness and a risk factor for aspiration and pneumonia after surgery (7). The various causes of RLNP include thermal damage, compression, ischemia, and towing among the various causes of RLNP. The reported rate of RLNP during esophagectomy ranges from 14.0 to 76.2% (8–11). Protection of the RLN during surgery is important in preventing complications and aiding in patient recovery. Intra-operative nerve monitoring (IONM), which can identify nerves and is widely utilized in thyroid surgery, has been demonstrated as a good approach for protecting RLN (12–15). Recent studies examining the utility of IONM in esophagectomy reported its feasibility and efficacy in protecting the RLN (16–20). However, studies focusing on the utility of IONM as a method are limited and the number of patients included in these studies remains low. Therefore, we performed a systematic review and meta-analysis to elucidate the impact of IONM on protecting the RLN during thoracoscopic esophagectomy for esophageal cancer.

## Materials and Methods

### Search Strategy

A literature search of PubMed, EMBASE, EBSCO, Web of Knowledge, and Cochrane Library until June 4, 2021 was conducted using the terms “(nerve monitoring OR neuromonitoring OR neural monitoring OR recurrent laryngeal nerve) AND (esophagectomy OR esophageal).” **Patients who were diagnosed with esophagus cancer and underwent thoracoscopic esophagectomy were included for study. The outcome of RLNP were compared in the IONM and non-IONM group. Studies must be retrospective or prospective case control studies**.

The inclusion criteria were studies which included patients who underwent thoracoscopic esophagectomy for esophageal cancer and compared patients evaluated with IONM (IONM group) to those not evaluated with IONM (non-IONM group) and reported the outcome of RLNP. Following were the exclusion criteria: (1) articles using duplicated data from the same study, (2) studies that did not directly compare between IONM and non-IONM, (3) articles with only an abstract and no full text, and (4) articles not in English. Relevant articles were also identified through the reference lists of relevant review articles.

### Data Extraction

Data were independently retrieved from the identified studies by two authors (XW and HG). Consensus was reached by discussion in cases of disagreement. The following data were retrieved from the studies: first author; country; publication year; study design; number of patients; sex; smoking history; location of tumor; pathological type; prior therapy; cancer stage according to the American Joint Committee on Cancer guidelines; incidence of RLNP; complications including aspiration, pneumonia, and hoarseness; number of dissected lymph nodes; operation time; time of thoracic procedure; and length of hospital stay.

### Quality Assessment

The quality of included studies were assessed using the Newcastle–Ottawa scale, and a Newcastle–Ottawa scale score of >6 were used to define high-quality studies. The assessment was independently performed by two authors (QH and YY), and a third author (BC) was consulted to settle disagreements if necessary.

### Statistical Analysis

All statistical analyses were performed using the Review manager software (version 5.3). Between-study heterogeneity was calculated using Higgins' *I*^2^ statistics (21). Pooled results with an *I*^2^ > 50% were considered to exhibit a high degree of heterogeneity, and the random-effects model was used to estimate. A fixed-effects model was used in studies with a low degree of heterogeneity. Sensitivity analysis was performed in the presence of a high degree of heterogeneity. Pooled odds ratios (ORs) and the corresponding 95% confidence intervals (CIs) were estimated for dichotomous outcomes, and mean differences were estimated for continuous outcomes. The Mantel–Haenszel and inverse variance methods were used for the analysis of dichotomous and continuous outcomes, respectively. Publication bias was qualitatively estimated using funnel plots. Mean and standard deviation of samples were estimated using sample sizes, medians, ranges, and/or interquartile ranges, as described by Wan et al. (22). The overall effect was considered statistically significant with a two-sided *p* < 0.05.

## Results

### Search Results

**Figure 1:**
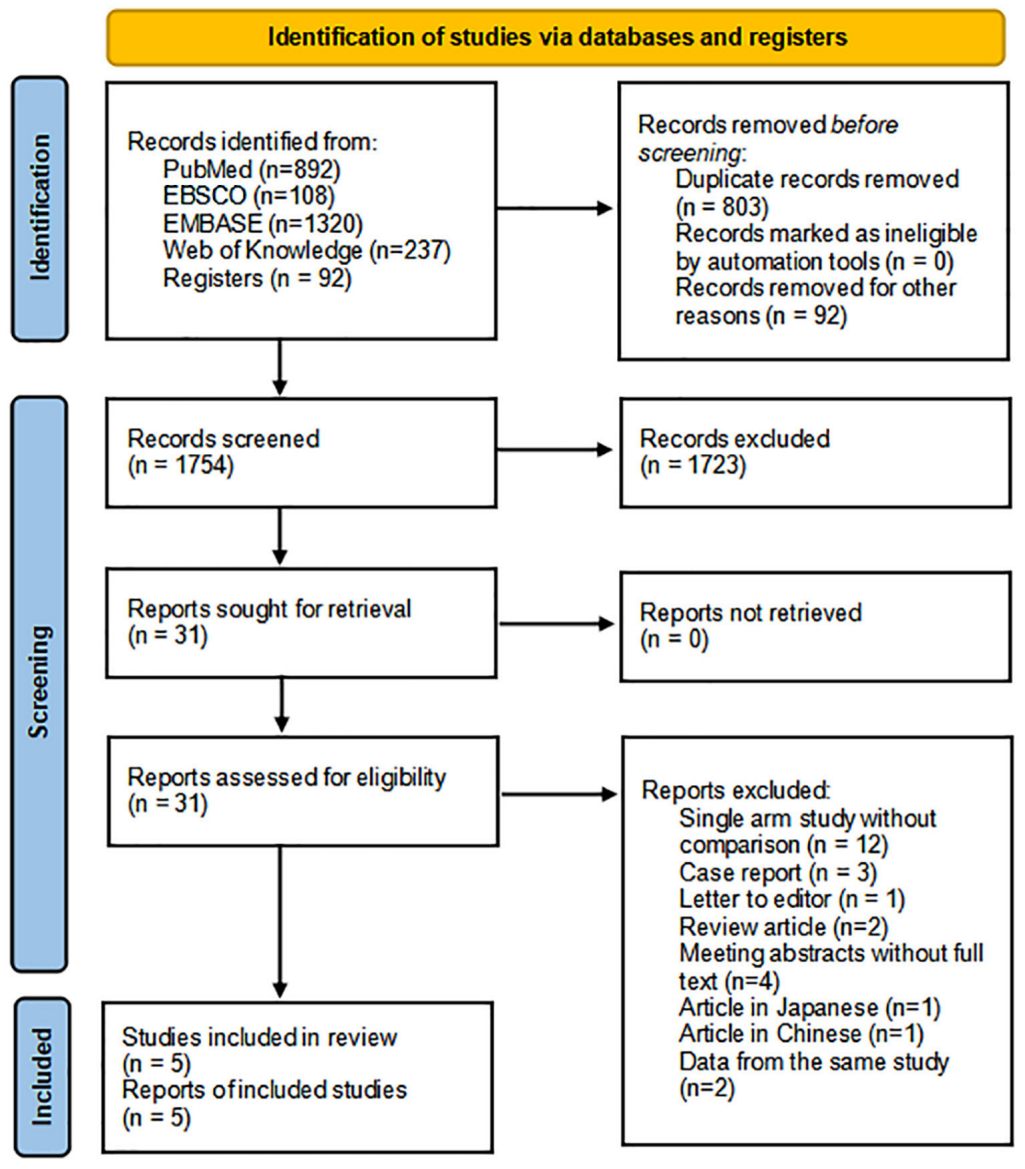
PRISMA flow diagram for study identification.

The literature search of the five electronic databases included in the study identified a total of 2,649 studies fulfilling the inclusion criteria. After preliminary screening, 31 studies were retrieved to assess eligibility. Of these, 12 studies which did not compare IONM with non-IONM (23–34). Three case reports on IONM (35–37), one letter to the editor (38), and two reviews (39, 40) were excluded. Additionally, four meeting abstracts lacking full texts (41–44), two studies which utilized data from the same study (45, 46), and two studies which were not in English (47, 48) were excluded. Finally, a total of five studies, including three retrospective cohort studies and two prospective cohort studies, comprising a total of 509 patients undergoing thoracoscopic esophagectomy for esophageal cancer were identified (16–20). In total, there were 240 and 269 patients who underwent surgery with and without IONM, respectively. The PRISMA flow diagram of study identification is presented in Figure 1.

### Patient Characteristics

The detailed baseline information of patients is summarized in Table 1. Briefly, the mean patient age ranged from 58.9 to 69.5 years in the non-IONM group and from 59.9 to 67.7 years in the IONM group. All studies reported tumor location, and most tumors were located in middle and lower esophagus. All but one of the studies reported the pathological tumor type, and the majority were squamous cell cancer. The percentage of patients who received therapy prior to surgery was not significantly different between the IONM and non-IONM groups in four of the five studies which reported that patients received therapy prior to surgery. All studies reported the cancer stage of patients according to the American Joint Committee on Cancer guidelines, and the cancer stage did not significantly differ between the IONM and non-IONM group.

**Table 1:** Baseline characteristics of patients.

**Study**	**Group**	**Hikage et al**.	**Fujimoto et al**.	**Zhong et al**.	**Kobayashi et al**.	**Takeda et al**.
Publication year		2016	2021	2014	2018	2020
Study design		Retrospective cohort	Prospective cohort	Prospective cohort	Retrospective cohort	Retrospective cohort
Country		Japan	Japan	China	Japan	Japan
Patients	Non-IONM	54	15	61	56	83
	IONM	54	17	54	31	84
Mean age (y)	Non-IONM	64.9 ± 6.8	69.5	58.9 ± 9.6	67.3 ± 5.9	65.7 ± 7.0
	IONM	67.1 ± 9.5	66.3	59.9 ± 9.0	66.5 ± 5.8	67.7 ± 8.4
Sex
Male	Non-IONM	41	15	49	44	72
	IONM	49	13	45	25	70
Smoking history	Non-IONM	45	11	40	44	/
	IONM	44	12	28	25	/
Location of tumor						
Ut	Non-IONM	3	1	/	6	16
Mt		29	11	46	23	41
Lt		16	3	17	25	26
Ae		6	0	/	2	0
Ut	IONM	8	4	/	5	21
Mt		25	8	42	10	38
Lt		13	4	12	16	25
Ae		8	1	/	0	0
Pathological type
SCC	Non-IONM	48	15	54	51	/
AC		4	0	3	2	/
Others		2	0	4	3	/
SCC	IONM	46	16	49	26	/
AC		5	1	2	3	/
Others		3	0	3	2	/
Prior therapy
Yes	Non-IONM	39	6	/	28	30
No	IONM	33	10	/	16	49^**^
AJCC stage
I	Non-IONM	21	5	18	28	35
II		15	2	19	18	17
III		17	7	24	9	20
IV		1	1	0	1	10
I	IONM	17	4	10	11	31
II		13	4	12	10	22
III		22	8	32	9	19
IV		2	1	0	1	10

*AC, adenocarcinoma; Ae, abdominal esophagus; AJCC, American Joint Committee on Cancer; IONM, intraoperative nerve monitoring; Lt, lower thoracic esophagus; Mt, middle thoracic esophagus; SCC, squamous cell carcinoma; Ut, upper thoracic esophagus*.

** *The chi-square test was used to compare two groups, and significance was set at a p < 0.05*.

### Outcome of RLNP

**Figure 2:**
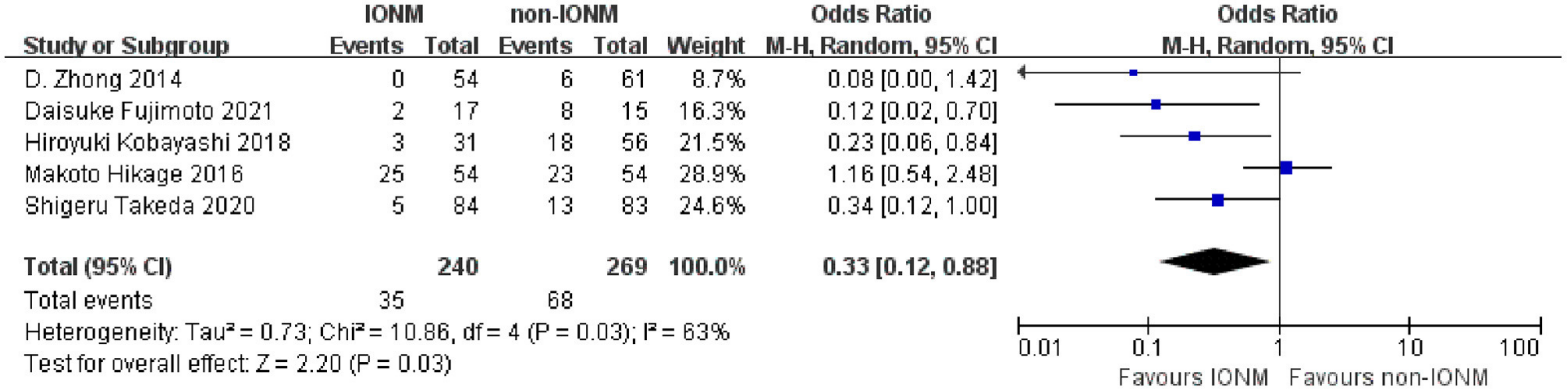
Forest plot showing RLNP rates after thoracoscopic esophagectomy in the IONM and non-IONM groups.

All studies reported RLNP as a surgical outcome. RLNP occurred in both the IONM and non-IONM groups in four studies, except for the study by Zhong et al. The detailed results of IONM are presented in Table 2. The rate of RLNP ranged from 0 to 46.3% in the IONM group and from 9.8 to 53.3% in the non-IONM group. The pooled analysis showed that IONM was associated with a significant reduction in the rate of RLNP after thoracoscopic esophagectomy (OR 0.33, 95%CI 0.12–0.88, *p* < 0.05, Figure 2). Three studies reported the correlation between signal loss and postoperative RLNP in the IONM group. The ranges of sensitivity, specificity, positive predictive value, and negative predictive value were 0–66.7%, 54.8–100%, 0–100%, and 68.0–96.6%, respectively.

**Table 2:** Results of IONM.

**Study**	**IONM signal loss**	**No**	**Yes**	**No**	**Yes**	**Sensitivity**	**Specificity**	**PPV**	**NPV**
	**Vocal cord motion**	**No**	**No**	**Yes**	**Yes**				
Hikage et al.^†^		4	0	43	7	0.0%	86.0%	0.0%	91.5%
Hikage et al.^‡^		8	15	17	14	65.2%	54.8%	51.7%	68.0%
Fujimoto et al.		/	/	/	/	/	/	/	/
Zhong et al.		/	/	/	/	/	/	/	/
Kobayashi et al.		2	1	28	0	66.7%	100.0%	100.0%	96.6%
Takeda et al.		9	9	60	6	50.0%	90.9%	60.0%	87.0%

*IONM, intraoperative nerve monitoring; PPV, positive predictive value; NPV, negative predictive value*.

† *right side RLN monitoring in thoracic procedure*.

‡ *left side RLN monitoring in thoracic procedure*.

*Sensitivity, number of IONM-positive cases (loss of signal) among all cases with RLN paralysis; Specificity, number of IONM-negative cases among all cases without RLN paralysis; PPV, percentage of cases with postoperative RLN paralysis among all cases with RLN paralysis estimated by IONM; NPV, percentage of cases without postoperative RLN paralysis among all cases without RLN paralysis estimated by IONM*.

### Lymph Node Dissection and Surgical Outcomes

**Figure 3:**
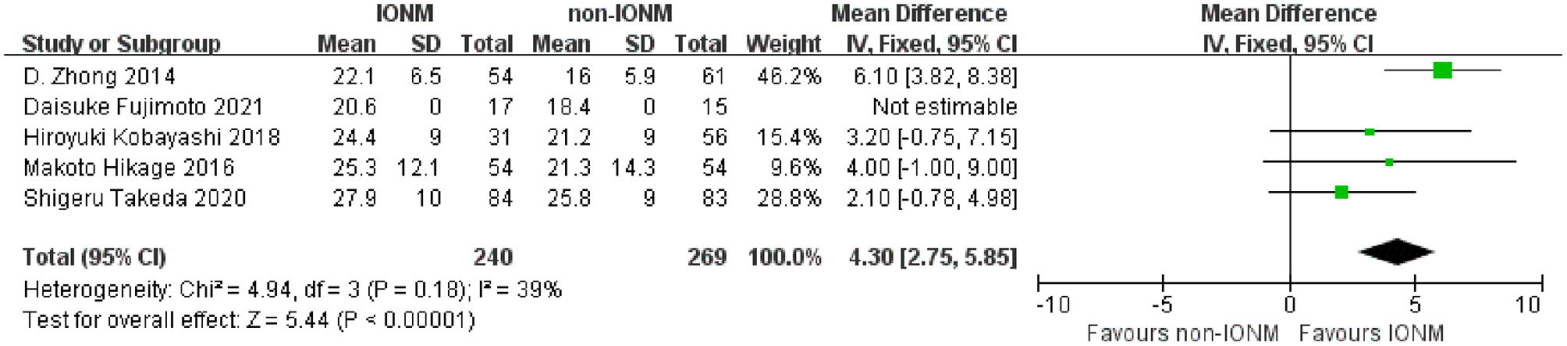
Forest plot of dissected mediastinal lymph nodes in the IONM and non-IONM groups.

**Figure 4:**
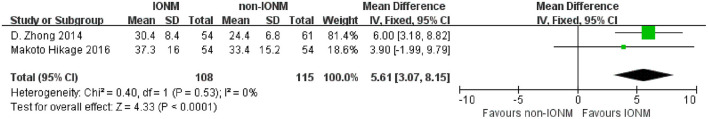
Forest plot of total dissected lymph nodes in the IONM and non-IONM groups.

**Figure 5:**
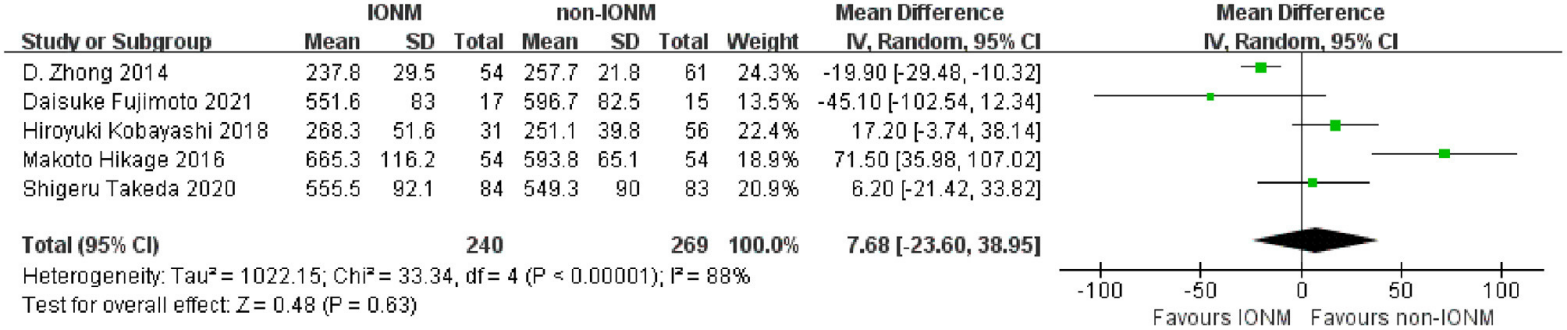
Forest plot of operation time in the IONM and non-IONM groups.

**Figure 6:**
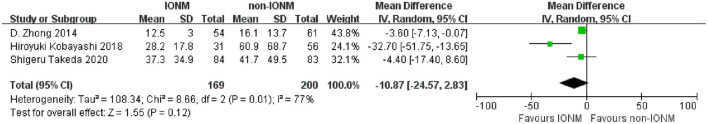
Forest plot of length of hospital stay in the IONM and non-IONM groups.

All studies reported the number of dissected mediastinal lymph nodes during surgery. The detailed information of lymph node dissection and surgical outcomes are presented in Table 3. Briefly, the mean number of dissected mediastinal lymph nodes ranged from 16.0 to 25.8 in the non-IONM group and from 20.6 to 27.9 in the IONM group. The pooled analysis revealed that there were significantly more dissected mediastinal lymph nodes in the IONM group than in the non-IONM group (mean difference 4.30, 95%CI 2.75–5.85, *p* < 0.001, Figure 3). Two studies reported the total number of dissected lymph nodes, including the mediastinal and abdominal lymph nodes. The pooled analysis showed that number of dissected lymph nodes was higher in the IONM group than in the non-IONM group (mean difference 5.61, 95%CI 3.07–8.15, *p* < 0.001, Figure 4). All studies reported the mean operation time, which ranged from 237.8 to 665.3 min in the IONM group and from 251.1 to 596.7 min in the non-IONM group. The pooled analysis showed that there was no significant difference in the operating time between the IONM and non-IONM groups (mean difference 7.68, 95%CI −23.60–38.95, *p* = 0.63, Figure 5). The mean length of hospital stay, which was reported in three studies, ranged from 12.5 to 37.3 days in the IONM group and from 16.1 to 60.9 days in the non-IONM group. The pooled analysis of the mean length of hospital stay revealed that there was no significant difference between the IONM and non-IONM groups (mean difference −10.87, 95%CI, −24.57–2.83, *p* = 0.12, Figure 6).

**Table 3:** Surgical outcomes.

**Study**	**Group**	**Hikage et al**.	**Fujimoto et al**.	**Zhong et al**.	**Kobayashi et al**.	**Takeda et al**.
RLNP
Left		19	8	/	11	9
Right		3	0	/	1	1
Bilateral	Non-IONM	1	0	/	6	3
No		31	7	55	38	70
Left		21	1	/	3	4
Right		2	0	/	0	0
Bilateral	IONM	2	1	/	0	1
No		29	15	54	28	79
Aspiration	Non-IONM	17	7	6	16	/
	IONM	24	2	0	2	/
Pneumonia	Non-IONM	4	6	15	11	19
	IONM	10	4	7	5	21
Hoarseness	Non-IONM	/	7	6	/	/
	IONM	/	2	0	3	/
No. of DMLN	Non-IONM	21.3 ± 14.3	18.4	16.0 ± 5.9	21.2 ± 9.0	25.8 ± 9.0
	IONM	25.3 ± 12.1	20.6	22.1 ± 6.5	24.4 ± 9.0	27.9 ± 10.0
No. of positive DMLN	Non-IONM	0.6 ± 1.5	/	1.4 ± 1.8	/	/
	IONM	1 ± 2.6	/	2.5 ± 2.6	/	/
No. of TDLN	Non-IONM	33.4 ± 15.2	/	24.4 ± 6.8	/	/
	IONM	37.3 ± 16.0	/	30.4 ± 8.4	/	/
No. of positive TDLN	Non-IONM	0.9 ± 2.4	/	1.7 ± 2.1	/	/
	IONM	2.8 ± 5.1	/	2.8 ± 3.0	/	/
Operative time (min)	Non-IONM	593.8 ± 65.1	596.7 ± 82.5	257.7 ± 21.8	251.1 ± 39.8	549.3 ± 90.0
	IONM	665.3 ± 116.2	551.6 ± 83.0	237.8 ± 29.5	268.3 ± 51.6	555.5 ± 92.1
Time of thoracic procedure	Non-IONM	/	262.3 ± 29.6	/	/	332.8 ± 35.0
	IONM	/	233.6 ± 28.7	/	/	312.6 ± 71.2
Length of stay (days)	Non-IONM	/	/	16.1 ± 13.7	60.9 ± 68.7	41.7 ± 49.5
	IONM	/	/	12.5 ± 3.0	28.2 ± 17.8	37.3 ± 34.9

*IONM, intraoperative nerve monitoring; RLNP, recurrent laryngeal nerve palsy; DMLN, dissected mediastinal lymph nodes, TDLN, total dissected lymph nodes including mediastinal and intraabdominal lymph nodes*.

### Complications

**Figure 7:**
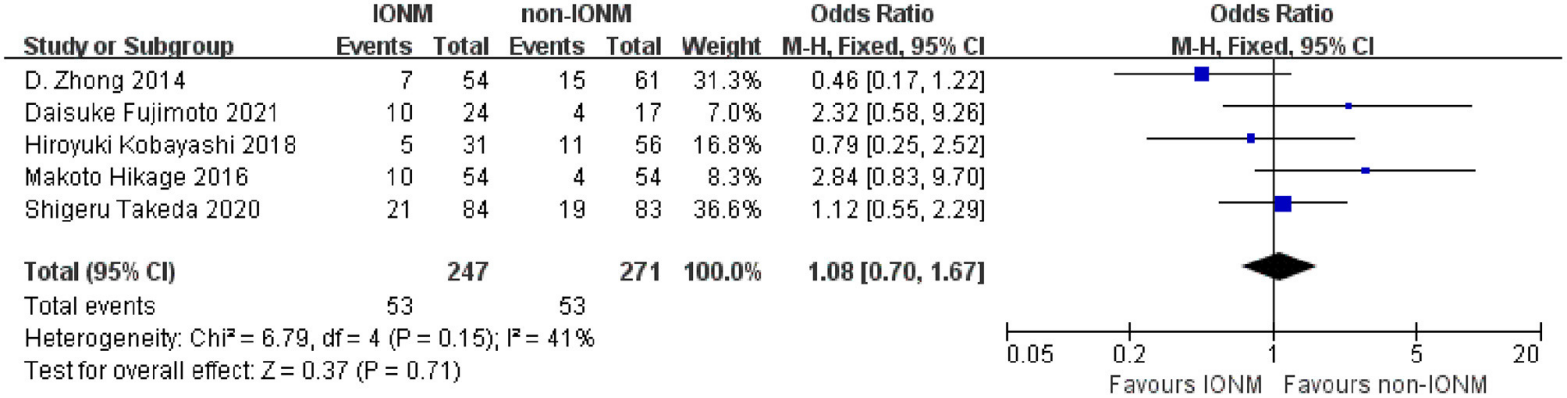
Forest plot of pneumonia in the IONM and non-IONM groups.

**Figure 8:**
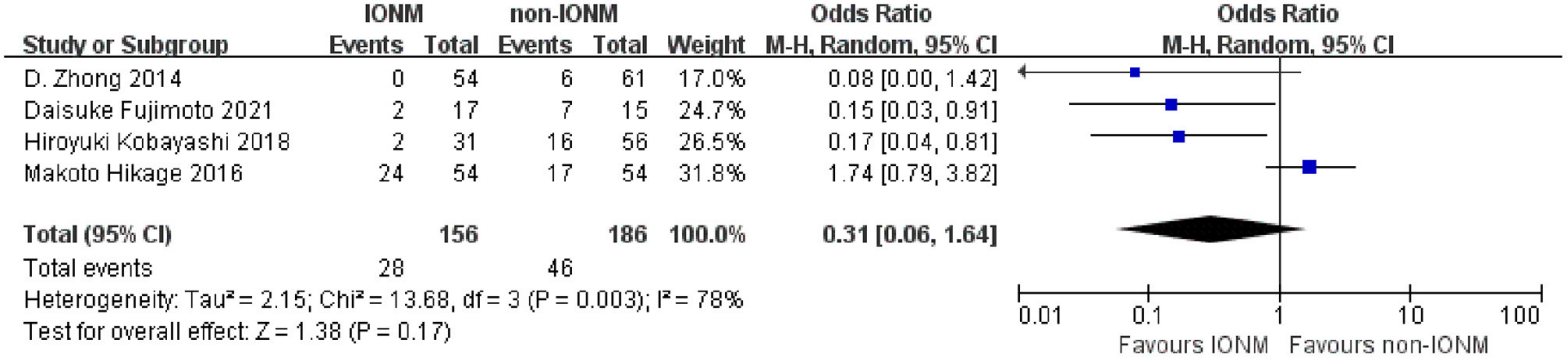
Forest plot of aspiration in the IONM and non-IONM groups.

**Figure 9:**
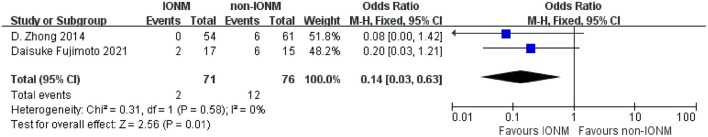
Forest plot of hoarseness in the IONM and non-IONM groups.

All studies reported the rate of pneumonia after surgery, which ranged from 7.4 to 40% in the non-IONM group and from 13.0 to 25.0% in the IONM group. The pooled analysis of pneumonia revealed no significant difference in the rate of pneumonia between the IONM and non-IONM groups (OR 1.08, 95%CI 0.70–1.67, *p* = 0.71, Figure 7). Four studies reported the rate of aspiration after surgery, which ranged from 9.8 to 46.7% in the non-IONM group and from 0 to 44.4% in IONM group. The pooled analysis showed that there was no significant difference in the rate of aspiration after surgery between the IONM and non-IONM groups (OR 0.31, 95%CI 0.06–1.64, *p* = 0.17, Figure 8). Two studies reported the rate of hoarseness after surgery, which ranged from 9.8 to 40% in the non-IONM group and from 0 to 11.8% in the IONM group. The pooled analysis showed that the rate of hoarseness was significantly lower in the IONM group than in the non-IONM group (OR 0.14, 95%CI 0.03–0.63, *p* < 0.05, Figure 9).

### Heterogeneity and Sensitivity Analysis

We observed significant heterogeneity in the data on rates of RLNP, aspiration, operation time, and length of hospital stay (*I*^2^, 63, 78, 88, and 77%, respectively). Therefore, we performed sensitivity analysis by excluding each study and recalculated pooled ORs with 95%CIs. After excluding the study by Hikage et al., the ORs for RLNP and aspiration were in favor of IONM (OR 0.23, 95%CI 0.11–0.48, *I*^2^ = 0%, and OR 0.15, 95%CI, 0.05–0.44, *I*^2^ = 0, respectively). The rate of operation time did not exhibit a significant difference after excluding each study. However, after excluding the study by Kobayashi et al., the OR for length of hospital stay was in favor of IONM (mean difference −3.65, 95%CI, −7.06–−0.25, *p* < 0.05, *I*^2^ = 0%).

### Publication Bias

The funnel plot for RLNP (Figure 1) revealed that the studies exhibited an almost symmetrical distribution on each side, and an obvious publication bias was not observed.

### Quality Assessment

The quality assessment of the studies is shown in Table 4. Briefly, all studies had a Newcastle–Ottawa scale score of nine, which indicated good methodological quality. No study was excluded because of low quality.

**Table 4:** Quality assessment of the nonrandomized studies using the Newcastle-Ottawa scale.

**Publication**	**Study**	**Selection**				**Comparability (Based**	**Outcome**			
**year**						**on design**				
						**and analysis)**				
		**Representativeness**	**Selection of**	**Ascertainment**	**Outcome of**		**Assessment**	**Follow-up**	**Adequacy of**	**Total**
		**of exposed**	**non-exposed**	**of exposure**	**interest**		**outcome**	**long enough**	**follow-up**	**score**
					**absent at**			**for outcomes**		
								**to occur**		
2016	Hikage et al.	1	1	1	1	2	1	1	1	9
2021	Fujimoto et al.	1	1	1	1	2	1	1	1	9
2014	Zhong et al.	1	1	1	1	2	1	1	1	9
2018	Kobayashi et al.	1	1	1	1	2	1	1	1	9
2020	Takeda et al.	1	1	1	1	2	1	1	1	9

## Discussion

To our knowledge, this is the first systematic review and meta-analysis examining the relationship between IONM and RLNP, which revealed that IONM could significantly reduce the rate of RLNP, improve lymphadenectomy, and reduce hoarseness after thoracoscopic esophagectomy for esophageal cancer. But we did not observe significant differences in operation time, aspiration, pneumonia, and length of hospital stay between the patients evaluated with IONM and those who were not evaluated by IONM during surgery.

The rate of RLNP was significantly lower in the IONM group than in the non-IONM group, although there was a decent degree of heterogeneity. After excluding the study by Hikage et al. (19), the rate of RLNP remained significantly lower in the IONM group without heterogeneity among the studies, which might be explained by the low sensitivity of IONM. In the study by Hikage et al., the surgeon was not familiar with the manipulation of the nerve monitoring system in the first 10 cases and IONM was discontinued in almost 30% of all patients in the IONM group for a variety of reasons, which might be associated with the lack of reduction in RLNP rate in that study.

The number of dissected mediastinal lymph nodes and all dissected lymph nodes, including mediastinal and abdominal lymph nodes, were significantly higher in the IONM group compared with the non-IONM group. IONM during surgery facilitated the radical dissection of lymph nodes along bilateral RLNs without high risk of injury to the RLN. The ratio of positive total dissected lymph nodes was significantly higher in the IONM group in the study by Zhong et al. (17). Lymph node metastasis is a risk factor for survival (49, 50); therefore, radical lymphadenectomy is necessary for accurate cancer staging and improving survival, which was demonstrated in the study.

In the present meta-analysis, the incidence of aspiration was not significantly lower in the IONM group compared with the non-IONM group. Studies previously reported the association of aspiration with RLNP after surgery for esophageal cancer (7, 10, 51). The reduction in the rate of RLNP after surgery in patients receiving IONM might be partially responsible for the reduced rate of aspiration. Conversely, there was also no significant difference in the rate of pneumonia between the IONM and non-IONM groups. In the study by Zhong et al. (17), the rate of postoperative pneumonia was significantly lower in the IONM group compared with the non-IONM group, which might have been due to the higher percentage of patients with smoking history in the non-IONM group; history of smoking is a proven risk factor for postoperative pneumonia (52). Therefore, large-scale studies are warranted to clarify the association between IONM and postoperative pneumonia.

The operation time was not significantly different between the IONM and non-IONM groups, which might be due to radical lymphadenectomy and nerve identification using electrodes during both the thoracic and cervical procedures.

The survival rates of patients in the IONM and non-IONM groups were present only in the study by Zhong et al., in which the Kaplan–Meier analysis indicated that survival was significantly better in the IONM group. This outcome might be associated with the use of radical and complete lymphadenectomy in the IONM group; this approach can identify the metastasis lymph nodes and allows for accurate clinical staging. Of course, additional studies are warranted to elucidate the association between IONM and survival.

To our knowledge, this is the first systematic review and meta-analysis focusing on the impact of IONM on protecting RLN during thoracoscopic esophagectomy for esophageal cancer. However, the present study has several limitations that should be acknowledged. First, none of the studies included in the analysis were randomized. Second, only studies published in English were included and studies reported in other language might have been missed. Third, the small number of studies and patients included in the meta-analysis might not be an accurate representation to assess the impact of IONM on RLNP. Fourth, there was a decent degree of heterogeneity in the current study results, which should be interpreted with caution. Finally, other factors such as ethnicity, **prior therapy** and surgical technique might also have an impact on RLNP.

In conclusion, the results of the present systematic review and meta-analysis suggested that IONM was associated with lower rates of RLNP and hoarseness and more effective lymphadenectomy after thoracoscopic esophagectomy for esophageal cancer. However, IONM was not associated with a significant benefit in preventing pneumonia and aspiration or reducing operation time and length of hospital stay.

## Data Availability Statement

The original contributions presented in the study are included in the article/supplementary material, further inquiries can be directed to the corresponding author/s.

## Author Contributions

XW: drafted the manuscript. BC: revised the manuscript. HG and XW: extracted the data and XW did the analysis. QH and YY: assessed the quality of the included studies. All authors contributed to the article and approved the submitted version.

## Funding

The work was supported by Taizhou Municipal Science and Technology Bureau (No. 1801ky09) and the Medical Health Science and Technology Project of Zhejiang Provincial Health Commission (No. 2019KY773). The funding has no role in the design of the study and collection, analysis, interpretation of data and writing of the manuscript.

## Conflict of Interest

The authors declare that the research was conducted in the absence of any commercial or financial relationships that could be construed as a potential conflict of interest.

## Publisher's Note

All claims expressed in this article are solely those of the authors and do not necessarily represent those of their affiliated organizations, or those of the publisher, the editors and the reviewers. Any product that may be evaluated in this article, or claim that may be made by its manufacturer, is not guaranteed or endorsed by the publisher.
